# The Impact of *TRIM67* Knockout on Early Intestinal Antimicrobial Capacity in Mice Infected with *Salmonella enterica* serovar Typhimurium ATCC 14028

**DOI:** 10.3390/microorganisms13061267

**Published:** 2025-05-29

**Authors:** Xinyue Zhang, Qinyuan Li, Tingting Zhang, Lanlan Jia, Wentao Liu, Chao Huang, Zhengli Chen, Qihui Luo

**Affiliations:** 1 Laboratory of Experimental Animal Disease Model, College of Veterinary Medicine, Sichuan Agricultural University, Chengdu 611130, China; sauxinyuezhang@163.com (X.Z.); rabbit84526@163.com (Q.L.); 19136342373@163.com (T.Z.); 2 Key Laboratory of Animal Disease and Human Health of Sichuan Province, College of Veterinary Medicine, Chengdu 611130, China; jialanlan2015@163.com (L.J.); liuwt1986@126.com (W.L.); huangchao@sicau.edu.cn (C.H.)

**Keywords:** *TRIM67*, *Salmonella enterica* serovar Typhimurium, macrophage polarization, NLRP3

## Abstract

*Salmonella enterica* serovar Typhimurium (*S.* Typhimurium) is an intracellular pathogen that survives and replicates within host cells. Macrophages, key immune cells in infection defense, play a vital role in pathogen clearance through polarization (M1/M2) and NLRP3 inflammasome activation. While *TRIM67* regulates macrophage recruitment in the liver, its role in *S.* Typhimurium infection remains unclear. In this study, a *S.* Typhimurium infection model was established by orally infecting streptomycin-pretreated *TRIM67* WT and KO mice with 1 × 10^9^ CFU of *S.* Typhimurium. *TRIM67* expression in the ileum, colon, mesenteric lymph nodes (MLNs), and peritoneal macrophages (PMs) was assessed via qRT-PCR and Western blotting. Histopathological changes were analyzed using HE and PAS staining. IHC staining, flow cytometry (FCM), qRT-PCR, and Western blotting were used to evaluate *TRIM67* knockout effects on macrophage recruitment, polarization, and NLRP3 inflammasome activation. In vitro, PMs were infected with *S.* Typhimurium (MOI 1:20), and *TRIM67*’s role in macrophage polarization and NLRP3 activation was validated. *S.* Typhimurium infection significantly upregulated *TRIM67* in the ileum, colon, and MLN. *TRIM67* knockout reduced intestinal inflammatory cell infiltration but worsened goblet cell loss and impaired digestion. Bacterial load assays revealed weakened pathogen clearance, leading to weight loss and increased mortality. *TRIM67* knockout inhibited intestinal macrophage recruitment, M1 polarization in MLN, and NLRP3 activation. In vitro, *TRIM67* knockout increased PMs’ intracellular bacterial load and suppressed NLRP3, caspase-1, and IL-1β expression. *TRIM67* knockout impairs the host’s ability to clear *S.* Typhimurium by inhibiting M1 macrophage polarization and NLRP3 inflammasome activation.

## 1. Introduction

Tripartite motif (TRIM) family proteins, characterized by their E3 ubiquitin ligase activity, play crucial roles in innate immunity, cell proliferation, and apoptosis [1,2,3,4,5]. Recent studies have shown that several TRIM family members, such as TRIM28 and TRIM31, regulate the assembly, activation, and degradation of the NLRP3 inflammasome, thereby modulating inflammatory responses [6,7,8]. *TRIM67*, a key member of the TRIM family, has been implicated in macrophage recruitment and inflammation regulation [9]. However, its role in host defense against infections through NLRP3 inflammasome modulation remains unclear.

*Salmonella enterica* serovar Typhimurium (*S.* Typhimurium) is a significant enteric pathogen responsible for gastroenteritis and systemic infections, causing hundreds of millions of cases annually worldwide [10,11,12,13]. As a facultative intracellular pathogen, *S.* Typhimurium can survive and replicate within macrophages [14,15,16]. Macrophage polarization (M1/M2) plays a pivotal role in infection control: M1 macrophages inhibit pathogen growth by releasing pro-inflammatory cytokines and reactive oxygen species, while M2 macrophages may provide a survival niche for pathogens [17]. Upon infection, *S.* Typhimurium is engulfed by macrophages, triggering local immune responses. After *S*. Typhimurium invades the mesenteric lymph node (MLN) via the intestine, the organ serves as a key barrier against the systemic spread of the pathogen, and its resident macrophages remove most of the migrating bacteria through phagocytosis, which is the core link in controlling the systemic spread of the pathogen and initiating adaptive immunity [18,19]. In this process, macrophages not only limit bacterial colonization to the spleen and liver, but also process antigens and present them to T and B cells, thereby activating a specific immune response to contain the spread of infection [20,21]. The absence of the MLN weakens host resistance, leading to an accelerated course of lethal infections and exacerbation of typhoid fever recurrence after antibiotic treatment, which further highlights the importance of an early antimicrobial response via the importance of macrophage-mediated pathogen clearance and immune activation in limiting secondary bacterial transmission [18,22].

The NLRP3 inflammasome is a critical component of host defense [23,24], whose activation induces the secretion of pro-inflammatory cytokines (e.g., IL-1β and IL-18) and pyroptosis, enhancing pathogen clearance [25,26,27]. Notably, NLRP3 inflammasome activation is closely linked to macrophage polarization: it not only promotes M1 macrophage-mediated pro-inflammatory responses but may also influence M2 polarization through IL-1β and IL-18 regulation [28,29,30,31]. However, whether *TRIM67* modulates NLRP3 inflammasome activity to defend against *S.* Typhimurium infection remains unknown.

Based on this background, we hypothesized that *TRIM67* plays a critical role in *S.* Typhimurium infection by regulating the NLRP3 inflammasome. This study employed *TRIM67* knockout mouse models and in vitro experiments to investigate the impact of *TRIM67* knockout on macrophage immune responses induced by *S*. Typhimurium, aiming to elucidate the molecular mechanisms underlying *TRIM67*-mediated host defense.

## 2. Materials and Methods

### 2.1. Animals

All animal experiments were conducted in strict compliance with the guidelines established by the Animal Care and Use Committee of Sichuan Agricultural University, ensuring ethical standards and welfare. The C57BL/6N mouse strain was utilized for this study. *TRIM67* gene knockout mice (*TRIM67*−/−, KO) were generated by Cyagen Biosciences (Suzhou, China) employing the CRISPR-Cas9 system to target exons 3, 4, and 5 of the *TRIM67* gene (Supplementary Figure S1). The guide RNA (gRNA) sequences designed for this purpose were as follows: gRNA1 (forward strand): TCTGGGTAGGTAACGGCTTCTGG; gRNA2 (reverse strand): CAGGCTCAAGGGGGTCTAGACGG. Genotyping was performed through PCR analysis of genomic DNA extracted from tail biopsies, using the following primer sets: for wild-type (WT) detection, forward 5′-GATGATAGCCATGTAATGCCCACC-3′ and reverse 5′-CCGTGATATGCTTGCCACAGGTTC-3′; for knockout (KO) identification, forward 5′-ATCAGAGATGGAGCAGACGCAG-3′ and reverse 5′-TTGATGGTTGGAGCCCTGC-3′ [32].

All mice were housed under specific pathogen-free (SPF) conditions and maintained at a controlled temperature of 20–22 °C, with a 12 h light/dark cycle, and relative humidity of 50–70%. Standard rodent chow and water were provided ad libitum.

### 2.2. Bacterial Strains

The bacterial suspension of *S.* Typhimurium (ATCC 14028s) was preserved long-term at −80 °C in an ultra-low temperature freezer within the Laboratory of Experimental Animal Disease Models, College of Veterinary Medicine, Sichuan Agricultural University (Chengdu, China). After picking the bacterial solution with an inoculating ring and using the three-zone delineation method, the bacteria were inoculated on SS (Salmonella Shigella) agar plates and placed in a 5% CO_2_, 37 °C incubator for 12–16 h. Single colonies were picked and added to 30 mL of sterile LB broth and then cultured in a shaker at 180 r/min and 37 °C for 12–16 h before passing on, and after passing on for 3–4 generations, the bacterial solution was used for the experiment. The bacteria were incubated in LB broth until the logarithmic growth phase (OD600 = 0.6) and then centrifuged at 4000 rpm for 5 min. The supernatant was discarded, and the bacterial pellet was resuspended in sterile phosphate-buffered saline (PBS), and the concentration of the bacterial broth was determined by a linear regression equation of OD600 versus the concentration of the bacterial broth (Supplementary Figure S3).

### 2.3. Construction of a S. Typhimurium Infection Mouse Model

SPF-grade C57BL/6N wild-type (WT) mice (8 weeks old, male) were randomly divided into 2 groups: WT uninfected control group (WT-CON) and WT infected group (WT-SAL). SPF grade C57BL/6N *TRIM67* knockout (KO) mice (8 weeks old, male) were randomly divided into 2 groups: KO uninfected control group (KO-CON) and KO infected group (KO-SAL). One day prior to infection, all mice were fasted for 4 h with water withheld. Streptomycin administered prior to infection reduces the gut microbiota and promotes Salmonella colonization. Therefore, both the control and infected groups were orally administered 0.1 mL (200 mg/mL) of streptomycin (Beyotime, Shanghai, China) via gavage. Food and water were restored 4 h post-administration. On the following day, mice were fasted again for 4 h, after which the control groups received 0.2 mL of sterile PBS, while the infected groups were administered 0.2 mL of a bacterial suspension (5 × 10^9^ CFU mL^−1^) via gavage [33]. Three days after infection, mice were anesthetized by intraperitoneal injection of 10% chloral hydrate (0.4 mL/100 g), and the ileum, colon, and mesenteric lymph nodes (MLNs) were collected aseptically as described in Supplementary Figure S4. A total of 38 WT mice and 41 KO mice were used in this experiment. Among them, 8 WT mice and 8 KO mice were used, with WT mice randomly divided into two groups (WT-CON, WT-SAL) and KO mice randomly divided into two groups (KO-CON, KO-SAL), 4 mice per group, for qRT-PCR, WB, HE, PAS, and IHC assays; 10 WT mice and 13 KO mice were used for survival rate analysis; 8 WT mice and 8 KO mice were used, with WT mice randomly divided into two groups (WT-CON, WT-SAL) and KO mice randomly divided into two groups (KO-CON, KO-SAL), 4 mice per group, for bacterial load testing; 6 WT mice and 6 KO mice were used, with WT mice randomly divided into two groups (WT-CON, WT-SAL) and KO mice randomly divided into two groups (KO-CON, KO-SAL), 3 mice per group, for flow cytometry assays; 6 WT mice and 6 KO mice were used for peritoneal macrophage isolation experiments.

### 2.4. Histological Analysis

Fresh colon tissues were fixed in 4% paraformaldehyde (PFA) at 4 °C for 24 h. The fixed samples were then embedded in paraffin and sectioned at a thickness of 5 μm. Following deparaffinization and rehydration, hematoxylin and eosin (H&E) staining was performed according to the manufacturer’s instructions (G1120 for H&E, Solarbio, Beijing, China). Goblet cells were stained using the Periodic Acid-Schiff (PAS) Staining Kit (including hematoxylin) (G1281, Solarbio, Beijing, China) following the provided protocol. Finally, the sections were mounted with neutral resin and imaged under a microscope (BX61VS, Olympus, Tokyo, Japan). The inflammatory cell infiltration score was determined based on the scoring criteria outlined in Table 1. There were three samples per group; three tissue sections were analyzed for each sample; three 200× fields of view were taken for each section; a field of view was scored three times, and the average of the three scores was taken.

### 2.5. qRT-PCR

Total RNA from colon tissues, MLN, and cells was extracted using the Animal Total RNA Extraction Kit (RE-03014, Foregene, Chengdu, China). Reverse transcription was performed using the RT EasyTM II Kit (with gDNase) (RT-01032, Foregene, Chengdu, China). qRT-PCR was conducted using the Real-time PCR EasyTM-SYBR Green I Kit (QP-01014, Foregene, Chengdu, China) on a Bio-Rad^®^ CFX96 PCR system (Bio-Rad, Hercules, CA, USA). Relative changes in gene expression were calculated by the ΔΔCt method. β-actin was used as the internal control for normalizing gene expression levels. Primers used for tissues and cells are listed in Table 2.

### 2.6. Western Blot

MLN proteins were extracted with RIPA lysis buffer at a volume of 30–50 ug per well, concentrated by 5% SDS-PAGE (run at 75 V for 30 min), and then separated by 10% SDS-PAGE (run at 120 V for 45 min, and electrophoresis was stopped until bromophenol blue reached the gel front). This was followed by transfer to a PVDF membrane (Millipore, Darmstadt, Germany). The membrane was blocked with TBST containing 5% skimmed milk for 1 h and then incubated with primary antibody (Table 3) at 4 °C overnight. Custom *TRIM67* antibodies were generated by immunizing rabbits with a GST fusion protein (1:50) corresponding to exons 3–5 of the mouse *TRIM67* gene. After rinsing with TBST, the rabbits were incubated with secondary antibody (1:10,000; Absin, San Francisco, CA, USA) for 1 h at room temperature and then rinsed again. ChampChemi 910 (Sage Creation, Beijing, China) system was used to expose the membrane for image acquisition. Protein bands were analyzed for grayscale values by ImageJ 1.53a.

### 2.7. Immunohistochemical Staining

Immunohistochemical staining was performed using the SADB-POD kit (SA2002, Boster, Wuhan, China). Paraffin-embedded sections were deparaffinized, rehydrated, and treated with 3% H_2_O_2_ for 20 min to block endogenous peroxidase activity. For antigenic heat repair, sections were placed in an autoclave containing 1.5 L of sodium citrate buffer (pH 6.0), heated until outgassing was timed for 2 min, and cooled to room temperature. Then, sections were incubated overnight at 4 °C with primary antibodies diluted in PBS containing 1% donkey serum. Biotin-labeled secondary antibodies and SABC were then applied sequentially for 30 min each. Color development was performed using DAB, followed by hematoxylin counterstaining. Images were captured and analyzed using ImageJ 1.53a. Full details of the primary antibodies used are given in Table 3.

### 2.8. Isolation and Culture of Primary Peritoneal Macrophages from Mice

Primary peritoneal macrophages (PMs) were isolated from 8-week-old male WT/KO mice. Mice were intraperitoneally injected with 1 mL of 4% thioglycollate (Solarbio, Beijing, China) for three consecutive days. On the third day, mice were euthanized and sterilized in 75% ethanol for 3 min. The abdominal cavity was exposed by a midline incision, and 5 mL of DMEM (A4192101, Gibco, Waltham, MA, USA) was injected into the peritoneum. After gentle abdominal massage, the DMEM containing peritoneal macrophages was collected and transferred to a sterile 50 mL centrifuge tube. This process was repeated three times. The cell suspension was filtered through a 70 μm cell strainer (BD Falcon, Brookings, SD, USA) and centrifuged to obtain the cell pellet. Cells were cultured in DMEM supplemented with 10% fetal bovine serum (FBS, P30-3302, PAN, Hamburg, Germany) and 1% penicillin–streptomycin (P1400, Solarbio, Beijing, China). After 3 h of incubation at 37 °C with 5% CO_2_, non-adherent cells were removed, and adherent cells were cultured in complete medium [34,35].

### 2.9. In Vitro Infection

PMs were seeded into 12-well plates at 2 × 10^5^ per well. After adherence, cells were serum starved in DMEM for 2 h. The PMs were infected with the *S*. Typhimurium suspension from 2.2 at a multiplicity of infection (MOI) of 20. Meanwhile, the uninfected group treated PM with DMEM as a control. After 20 min of infection, the bacterial suspension was removed, and the cells were rinsed twice with PBS. The medium was changed to complete DMEM containing 50 μg/mL gentamicin. Cells were further cultured for 1 and 12 h. Cells cultured for 1 h were used for qRT-PCR and Western blot analysis, while cells cultured for 12 h were used for bacterial load assessment.

### 2.10. Bacterial Load Assay

The MLN, ileum, and colon were collected aseptically on ice, weighed, and homogenized to homogeneity in 1 mL of sterile PBS containing 0.3% Triton-X (Bain-Marie, Guangzhou, China) using a tissue homogenizer.

After removing the medium from the PM that had been incubated for 12 h, the cells were rinsed twice with 5 mL of PBS and then blown dry with PBS. The PBS-containing PM was placed in a 15 mL centrifuge tube and centrifuged at 1000 rpm for 3 min to obtain the cell precipitate. Cells were resuspended with 1 mL of sterile PBS containing 0.3% Triton-X and homogenized with a tissue homogenizer until uniform.

Homogenization was realized with sterile PBS diluted in a 1:10 gradient. Three appropriate gradient dilutions were taken for each sample, and 0.1 mL of the dilution was applied to SS agar (Haplocene, Qingdao, China) dishes, and three coated plate replicates were made for each gradient. The SS agar was inverted and incubated at 37 °C in an incubator for 24 h. The number of bacterial colonies in each plate was then counted [34].

### 2.11. Flow Cytometry

Tissue digestion: fresh MLN tissues were minced and washed with PBS. Tissue fragments were digested with 1 mL of type IV collagenase (41C21062, Worthington, Lakewood, NJ, USA; 50–200 U/mL in DMEM) at 37 °C for 10 min. Digestion was stopped with 3% FBS, and the cell suspension was filtered through a 70 μm strainer. Cells were centrifuged at 1000 rpm for 5 min, resuspended in PBS, and adjusted to 1 × 10^7^ cells mL^−1^.

Surface staining: for staining, 100 μL of cell suspension was mixed with 5 μL each of PerCP Anti-Mouse CD45 (RM04503, Novo Biotech, Beijing, China), FITC Anti-Mouse F4/80 (RMU0101, Novo Biotech, Beijing, China), and PE Anti-Mouse CD80 (RM08002, Novo Biotech, Beijing, China). After vortexing, samples were incubated in the dark for 30 min.

Permeabilization and intracellular staining: cells were permeabilized using the FOXP3/Transcription Factor Staining Buffer Set (00-5523-00, Invitrogen, Waltham, MA, USA) and stained with 5 μL PE/Cy7 Anti-Mouse CD206 (RM20604, Novo Biotech, Beijing, China) for 30 min at room temperature.

Data acquisition and analysis: after washing, samples were analyzed on a CytoFLEX flow cytometer (Beckman Coulter, Brea, CA, USA), and the data were processed using CytExpert 2.3 software.

### 2.12. Data Analysis

Data are presented as mean ± SEM. Statistical significance was determined using two-tailed Student’s t-test or one-way ANOVA in GraphPad Prism (version 8.0, USA). * *p* < 0.05, ** *p* < 0.01, and *** *p* < 0.001.

## 3. Results

### 3.1. S. Typhimurium Infection Upregulates TRIM67 Expression in Mouse Ileum, Colon, and MLN

**Figure 1:**
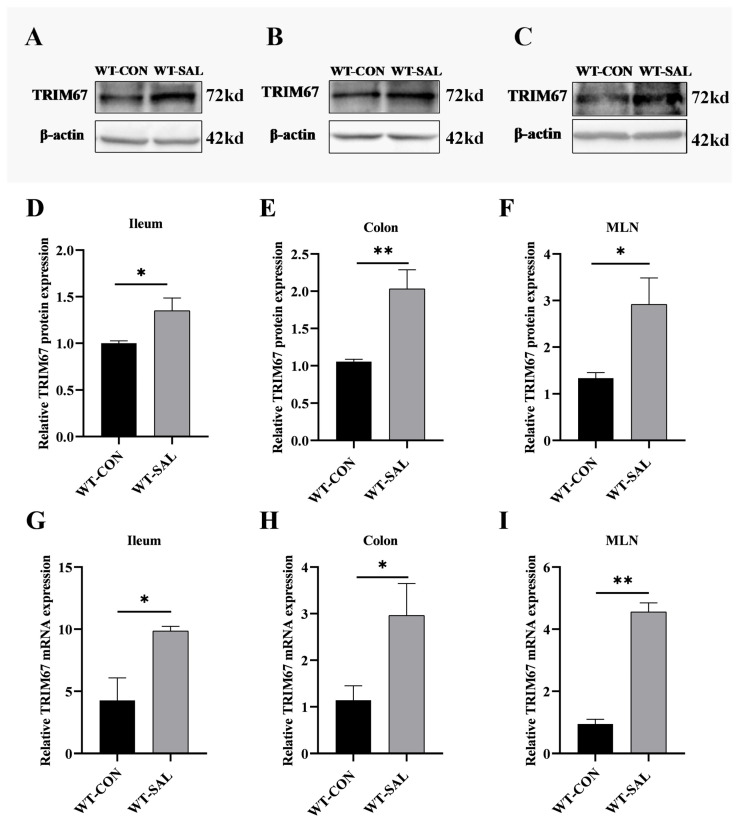
*S.* Typhimurium infection promotes *TRIM67* expression in the ileum, colon, and MLN. (**A**,**D**) Western blot and quantitative analysis of *TRIM67* protein expression in the ileum, *n* ≥ 3. (**B**,**E**) Western blot and quantitative analysis of *TRIM67* protein expression in the colon, *n* ≥ 3. (**C**,**F**) Western blot and quantitative analysis of *TRIM67* protein expression in the MLN, *n* ≥ 3. (**G**–**I**) Relative mRNA expression of *TRIM67* in the ileum, colon, and MLN, *n* ≥ 3. Error bars represent SEM; * *p* < 0.05, ** *p* < 0.01.

The ileum, colon, and mesenteric lymph nodes (MLNs) constitute a critical defense barrier against *S.* Typhimurium infection. To elucidate the role of *TRIM67* in *S.* Typhimurium infection, we first established a wild-type (WT) mouse model infected with *S.* Typhimurium. On day 3 post-infection, qRT-PCR and Western blotting were employed to evaluate *TRIM67* mRNA and protein expression levels in the ileum, colon, and MLN, which are the primary sites of *S.* Typhimurium colonization. The results revealed basal expression of *TRIM67* in the ileum, colon, and MLN of uninfected mice. In contrast, both *TRIM67* mRNA and protein levels were significantly upregulated in infected mice (*p* < 0.01 or 0.05) (Figure 1). These findings suggest that *S.* Typhimurium infection induces upregulation of *TRIM67*, thus emphasizing a possible role of *TRIM67* in *S.* Typhimurium infection of the ileum, colon, and MLN.

### 3.2. TRIM67 Knockout Exacerbates Weight Loss and Mortality in S. Typhimurium-Infected Mice

**Figure 2:**
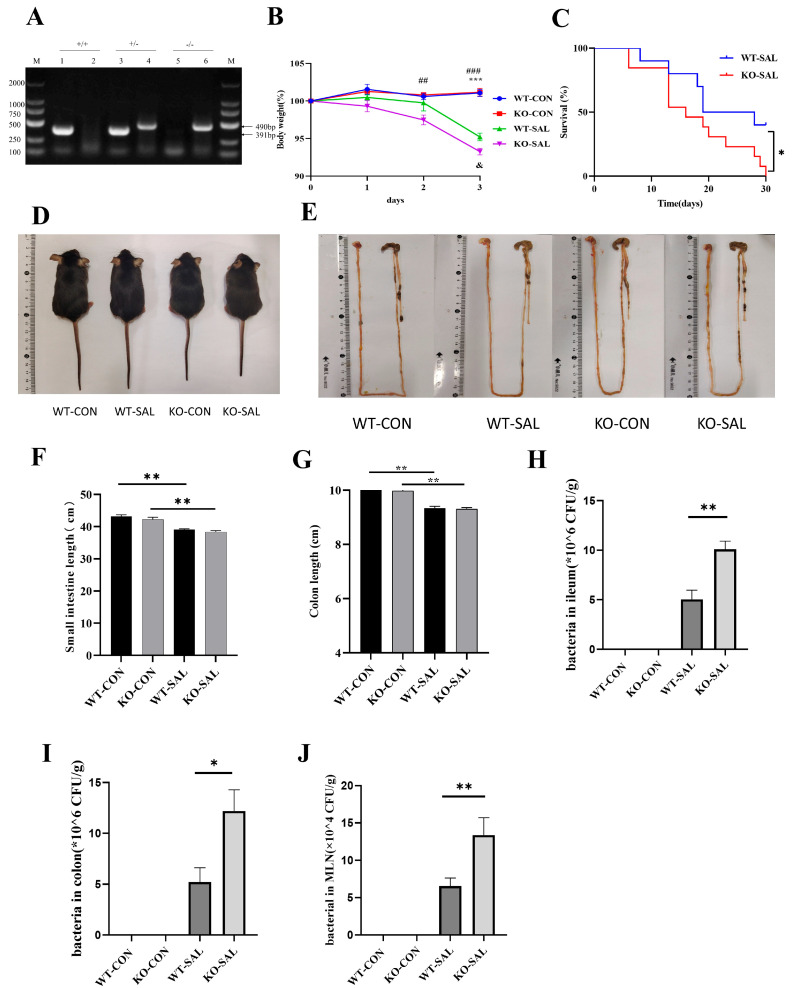
Impact of *TRIM67* knockout on mice infected with *S.* Typhimurium. (**A**) Genotyping of mice: lanes 1 and 2 identified as *TRIM67* +/+, lanes 3 and 4 as *TRIM67* +/−, and lanes 5 and 6 as *TRIM67* −/−. (**B**) Daily body weight changes during modeling, expressed as a percentage of initial weight. WT-CON vs. WT-SAL (*n* ≥ 3 and *** *p* < 0.001); KO-CON vs. KO-SAL (*n* ≥ 3, ## *p* < 0.01, and ### *p* < 0.001); WT-SAL vs. KO-SAL (*n* ≥ 3 and & *p* < 0.05). (**C**) Survival rate of mice during infection, *n* = 10, analyzed by Log-rank test. * *p* < 0.05. (**D**) Body size of mice. (**E**) Intestine size of mice. (**F**,**G**) Intestine and colon length of mice, *n* = 3. ** *p* < 0.01. (**H**–**J**) *S.* Typhimurium load in ileum, colon, and MLN, *n* = 4. * *p* < 0.05 and ** *p* < 0.01.

To explore *TRIM67*’s role in *S.* Typhimurium infection, we commissioned Cyagen Biosciences (Suzhou, China) to generate *TRIM67* knockout (KO) mice (Figure 2A, Supplementary Figure S5) and monitored weight changes and survival. Uninfected controls (WT-CON and KO-CON) showed steady weight gain, while infected groups (WT-SAL and KO-SAL) exhibited significant weight loss by days 2–3 (*p* < 0.01), with KO-SAL losing more weight than WT-SAL (*p* < 0.05) (Figure 2B). KO-SAL mice began dying by day 6, with 0% survival by day 30, compared to 40% in WT-SAL (Figure 2C). Body size did not differ among groups (*p* > 0.05) (Figure 2D). Infected groups had shorter small intestines (*p* < 0.01), with KO-SAL shorter than WT-SAL (*p* < 0.05) (Figure 2F). Colon length was also reduced in infected groups (*p* < 0.01), with KO-SAL shorter than WT-SAL (*p* < 0.01) (Figure 2G). KO-SAL mice had higher *S.* Typhimurium loads in the ileum, colon, and MLN than WT-SAL (Figure 2H–J). These results demonstrate that *TRIM67* knockout impairs *S.* Typhimurium clearance, underscoring its critical role in limiting infection in the ileum, colon, and MLN.

### 3.3. TRIM67 Knockout Inhibits Inflammatory Response and Exacerbates Intestinal Barrier Damage in the Gut of S. Typhimurium-Infected Mice

**Figure 3:**
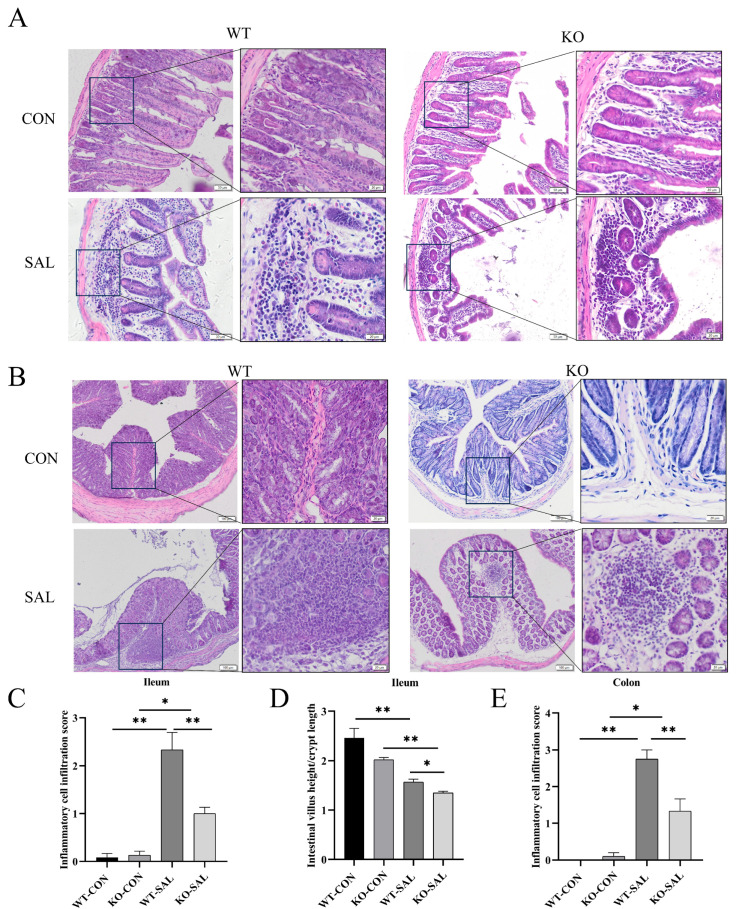
*TRIM67* knockout exacerbates intestinal inflammatory responses in mice infected with *S.* Typhimurium. (**A**) H&E staining of the ileum in mice, scale bars: 50 μm and 20 μm; (**B**) H&E staining of the colon in mice, scale bars: 100 μm and 20 μm; (**C**) Quantitative analysis of H&E staining in the ileum, *n* = 3; (**D**) villus height-to-crypt depth (V/C) ratio in the ileum, *n* = 3; (**E**) quantitative analysis of H&E staining in the colon, *n* = 3. * *p* < 0.05, ** *p* < 0.01.

To investigate the impact of *TRIM67* knockout on intestinal tissue structure in *S.* Typhimurium-infected mice, we performed histopathological analysis on the ileum and colon of each group. H&E staining revealed significant inflammatory cell infiltration in the ileum and colon of infected groups (WT-SAL and KO-SAL) compared to uninfected controls (WT-CON and KO-CON), with the degree of inflammatory cell infiltration was significantly weaker in KO-SAL than in WT-SAL (Figure 3A–C,E). The villus height-to-crypt depth ratio (V/C ratio), which reflects small intestinal digestive and absorptive function, was also assessed. Although the V/C ratio in KO-CON was slightly lower than in WT-CON, the difference was not significant. However, *S.* Typhimurium infection significantly reduced the V/C ratio in both WT-SAL and KO-SAL groups compared to uninfected controls (*p* < 0.01), with KO-SAL showing a further reduction compared to WT-SAL (*p* < 0.05) (Figure 3D). These findings indicate that *TRIM67* knockout exacerbates intestinal damage and impairs digestive and absorptive functions in *S.* Typhimurium-infected mice.

### 3.4. TRIM67 Regulates Intestinal Barrier Function by Modulating Goblet Cell Numbers in S. Typhimurium-Infected Mice

**Figure 4:**
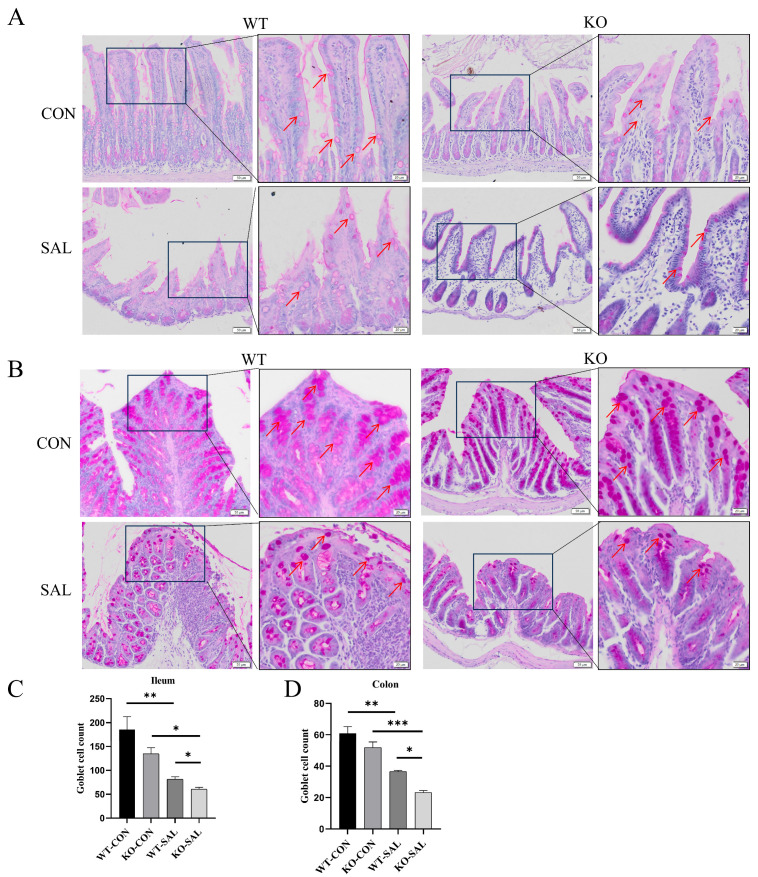
*TRIM67* knockout exacerbates goblet cell loss in the intestines of *S.* Typhimurium-infected mice. (**A**,**B**) PAS staining of the ileum and colon (scale bars: 50 μm and 20 μm). Red arrows indicate goblet cells. (**C**,**D**) Quantification of goblet cell numbers in the ileum and colon, *n* = 3. * *p* < 0.05; ** *p* < 0.01 and *** *p* < 0.001.

A previous study reported that *TRIM67* has an ameliorative effect on barrier function impairment induced by high-fat diet in obese mice [32]. To investigate the role of *TRIM67* in intestinal mucus and epithelial barrier function during *S.* Typhimurium infection, we assessed goblet cell numbers using PAS staining. The results showed that, compared to uninfected controls (WT-CON and KO-CON), the number of goblet cells in the ileum of infected groups (WT-SAL and KO-SAL) was significantly reduced (*p* < 0.01), with KO-SAL exhibiting a further decrease compared to WT-SAL (*p* < 0.05) (Figure 4A,D). In the colon, WT-SAL showed a significant reduction in goblet cells compared to WT-CON (*p* < 0.01), and KO-SAL also had fewer goblet cells than KO-CON (*p* < 0.05). Moreover, KO-SAL had fewer goblet cells than WT-SAL (*p* < 0.05) (Figure 4B,C). These findings indicate that *TRIM67* knockout exacerbates the intestinal barrier damage caused by *S.* Typhimurium infection. In summary, *TRIM67* knockout not only suppresses inflammatory cell infiltration in the intestines of infected mice but also further impairs intestinal barrier function.

### 3.5. TRIM67 Knockout Reduces NLRP3 Inflammasome Activation in S. Typhimurium-Infected Mice

**Figure 5:**
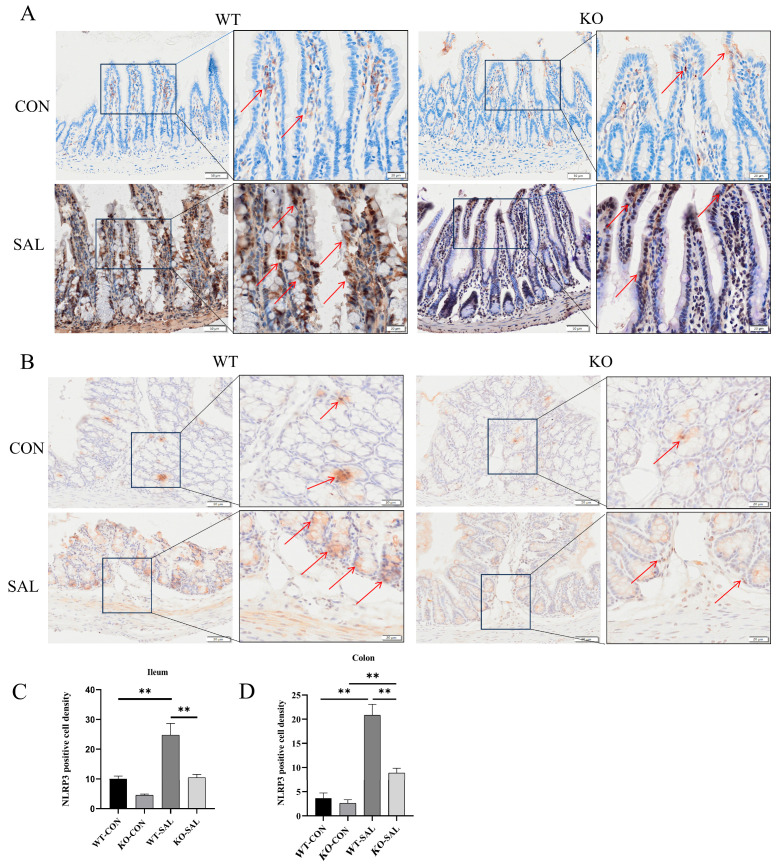
*TRIM67* inhibits NLRP3 inflammasome activation in the ileum and colon. (**A**) Immunohistochemical staining of NLRP3 in the ileum, and red arrows indicate NLRP3-positive cells (scale bars: 50 μm and 20 μm); (**B**) immunohistochemical staining of NLRP3 in the colon, and red arrows indicate NLRP3-positive cells (scale bars: 50 μm and 20 μm); (**C**) density of NLRP3-positive cells in the ileum, *n* = 3; (**D**) density of NLRP3-positive cells in the colon, *n* = 3. ** *p* < 0.01.

Decreased cuprocyte numbers are often accompanied by the suppression of NLRP3 inflammatory vesicle activity [36,37,38]. *S.* Typhimurium infection triggers robust inflammasome activation in macrophages. NLRP3 inflammasome activation promotes caspase-1 activation, leading to the maturation and release of inflammatory cytokines such as IL-1β and IL-18, thereby enhancing inflammatory responses to clear intracellular bacteria. To explore whether *TRIM67* regulates the NLRP3 inflammasome, we assessed NLRP3 expression in the ileum and colon of infected mice using immunohistochemical staining. The results showed that NLRP3 protein levels in the ileum and colon of infected groups (WT-SAL and KO-SAL) were significantly higher than in uninfected controls (WT-CON and KO-CON) (*p* < 0.01). However, KO-SAL exhibited lower NLRP3 levels compared to WT-SAL (*p* < 0.05) (Figure 5). These findings suggest that while *S.* Typhimurium infection upregulates NLRP3 inflammasome expression, *TRIM67* knockout suppresses this increase, highlighting its possible involvement in regulating the activity of NLRP3 inflammatory vesicles.

### 3.6. TRIM67 Knockout Reduces NLRP3 Inflammasome Activation in MLN of S. Typhimurium-Infected Mice

**Figure 6:**
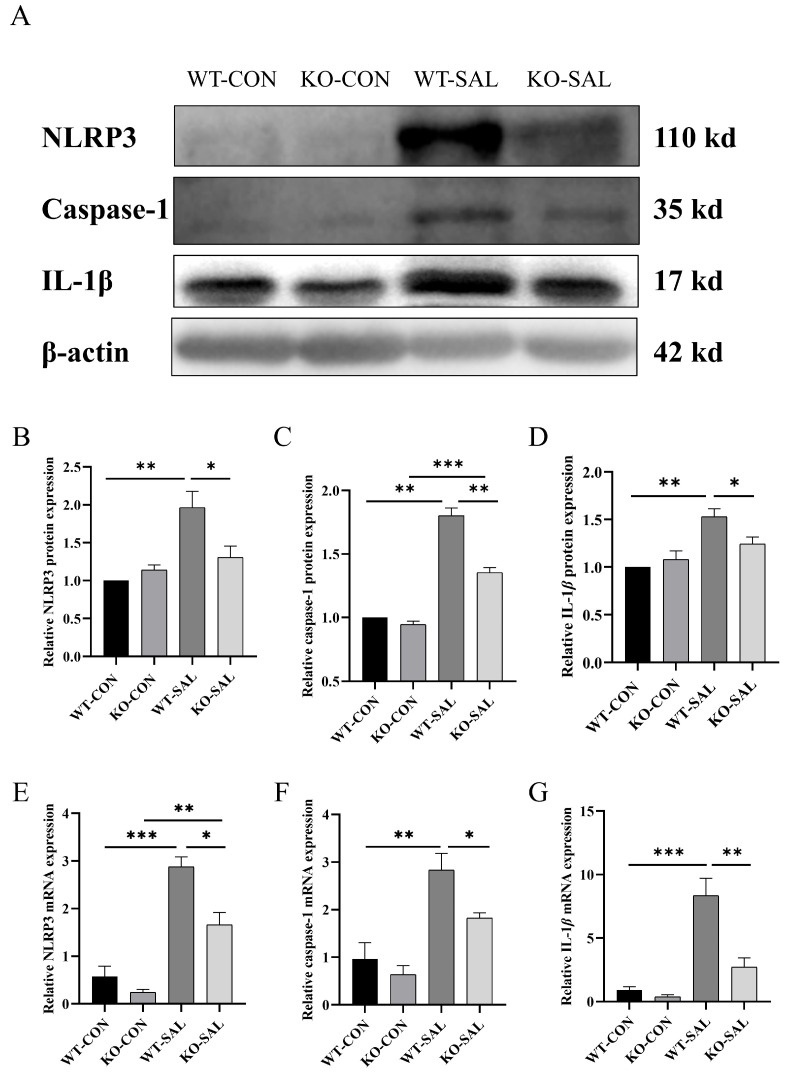
TRIM67 inhibits NLRP3 inflammasome activation in MLN. (**A**) Western blot analysis of NLRP3, caspase-1, IL-1β, and β-actin protein expression in the MLN; (**B**–**D**) relative protein expression levels of NLRP3, caspase-1, and IL-1β in the MLN, *n* = 3; (**E**–**G**) relative mRNA expression levels of NLRP3, caspase-1, and IL-1β in the MLN, *n* = 3. * *p* < 0.05, ** *p* < 0.01 and *** *p* < 0.001.

Activation of the NLRP3 inflammasome in the ileum and colon leads to the release of IL-1β and IL-18, which can reach the MLN via circulation or lymphatic drainage, further activating immune cells and linking local and systemic immune responses. To investigate whether *TRIM67* regulates NLRP3 inflammasome activity in the MLN, we assessed the expression levels of NLRP3, caspase-1, and IL-1β using Western blot and qRT-PCR. The results showed that, compared to uninfected controls (WT-CON and KO-CON), NLRP3 protein and mRNA levels in the MLN of WT-SAL mice were significantly elevated (*p* < 0.01), while KO-SAL mice exhibited significantly lower levels than WT-SAL (*p* < 0.05) (Figure 6A,B,E). Similarly, caspase-1 protein and mRNA levels in the MLN of WT-SAL mice were significantly higher than in uninfected controls (*p* < 0.01), but KO-SAL levels were significantly lower than WT-SAL (*p* < 0.05) (Figure 6A,C,F). Additionally, IL-1β protein and mRNA levels in the MLN of WT-SAL mice were significantly increased compared to uninfected controls (*p* < 0.01), while KO-SAL levels were significantly lower than WT-SAL (*p* < 0.05) (Figure 6A,D,G). These findings indicate that *S*. Typhimurium infection significantly induces NLRP3 inflammasome expression and activation in the MLN, while *TRIM67* knockout suppresses this process. This highlights the critical role of *TRIM67* in regulating NLRP3 inflammasome-mediated immune responses.

### 3.7. TRIM67 Knockout Inhibits Macrophage Recruitment and Polarization in the Intestine of S. Typhimurium-Infected Mice

**Figure 7:**
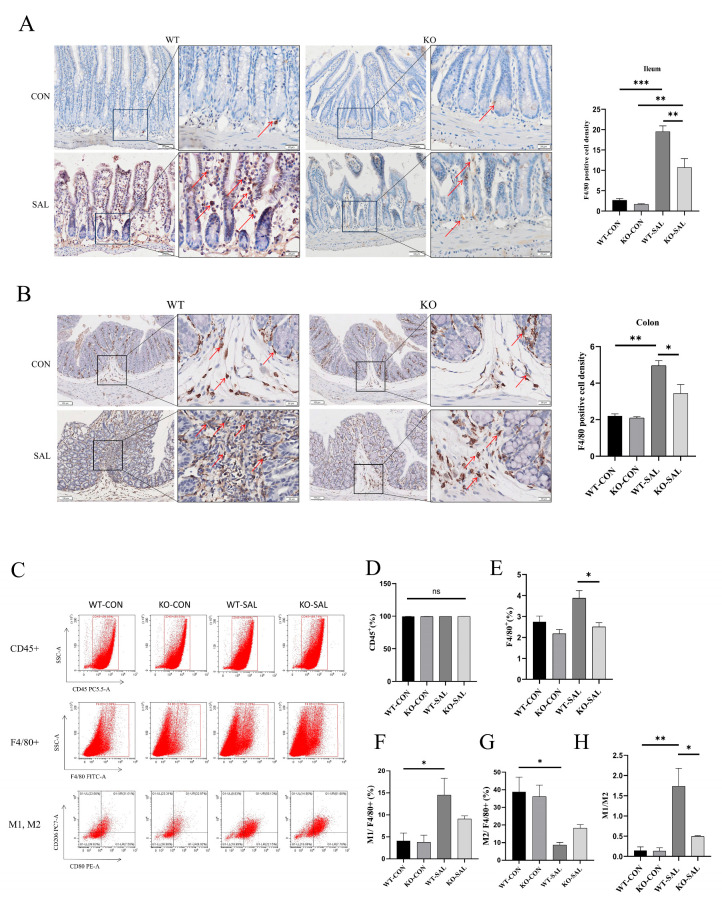
*TRIM67* knockout inhibits macrophage recruitment and polarization in the ileum and colon of *S*. Typhimurium-infected mice. (**A**) Immunohistochemical staining and quantification of the macrophage marker F4/80 in the ileum (scale bars: 50 μm and 20 μm). Red arrows indicate positive cells; (**B**) immunohistochemical staining and quantification of the macrophage marker F4/80 in the colon (scale bars: 100 μm and 20 μm). Red arrows indicate positive cells; (**C**–**H**) flow cytometry analysis and quantification of CD45^+^ cells, F4/80^+^ macrophage cells, M1 and M2 macrophage subtypes among F4/80+ cells in the MLN, and *n* = 3. * *p* < 0.05, ** *p* < 0.01 and *** *p* < 0.001.

Inhibition of intestinal macrophage recruitment by *TRIM67* knockdown was demonstrated by F4/80 immunohistochemical staining. Macrophage recruitment is crucial for maintaining macrophage populations at inflammatory and immune sites. TRIM family proteins are known to regulate macrophage-mediated immune responses, and *TRIM67* knockdown inhibits macrophage recruitment in the liver of mice with nonalcoholic fatty liver disease [9]. To determine whether *TRIM67* knockout similarly affects intestinal macrophage recruitment, we performed immunohistochemical staining for the macrophage marker F4/80 in the ileum and colon. Uninfected groups (WT-CON and KO-CON) exhibited a small number of resident macrophages, while the WT-SAL group showed significantly higher macrophage density compared to uninfected groups (*p* < 0.01) and the KO-SAL group (*p* < 0.05). Although macrophage density in the KO-SAL group was slightly elevated compared to uninfected groups, the difference was not statistically significant (Figure 7A,B). These findings indicate that *TRIM67* knockout inhibits macrophage recruitment in the ileum and colon during *S.* Typhimurium infection.

Evaluation of M1/M2 markers by flow cytometry demonstrated that *TRIM67* knockdown inhibited macrophage polarization to the M1 type in mesenteric lymph nodes. Macrophage polarization (M1/M2) plays a critical role in *S. Typhimurium* infection, with M1 macrophages inhibiting bacterial growth and M2 macrophages potentially promoting bacterial survival. To assess whether *TRIM67* influences macrophage polarization and antibacterial capacity, we analyzed MLN cells using flow cytometry. While CD45+ leukocyte counts did not differ significantly among groups, F4/80+ macrophage numbers in the KO-SAL group were significantly lower than in the WT-SAL group. The M1 (CD80 + CD206-)-to-M2 (CD206 + CD80-) ratio, reflecting polarization status, was higher in the WT-SAL group compared to the KO-SAL group (Figure 7C–H). Together, these results demonstrate that *TRIM67* knockout suppresses infection-induced macrophage recruitment and M1 polarization without affecting leukocyte numbers.

### 3.8. TRIM67 Knockout Inhibits Polarization of PMs and NLRP3 Inflammasome Activation in S. Typhimurium Infection

**Figure 8:**
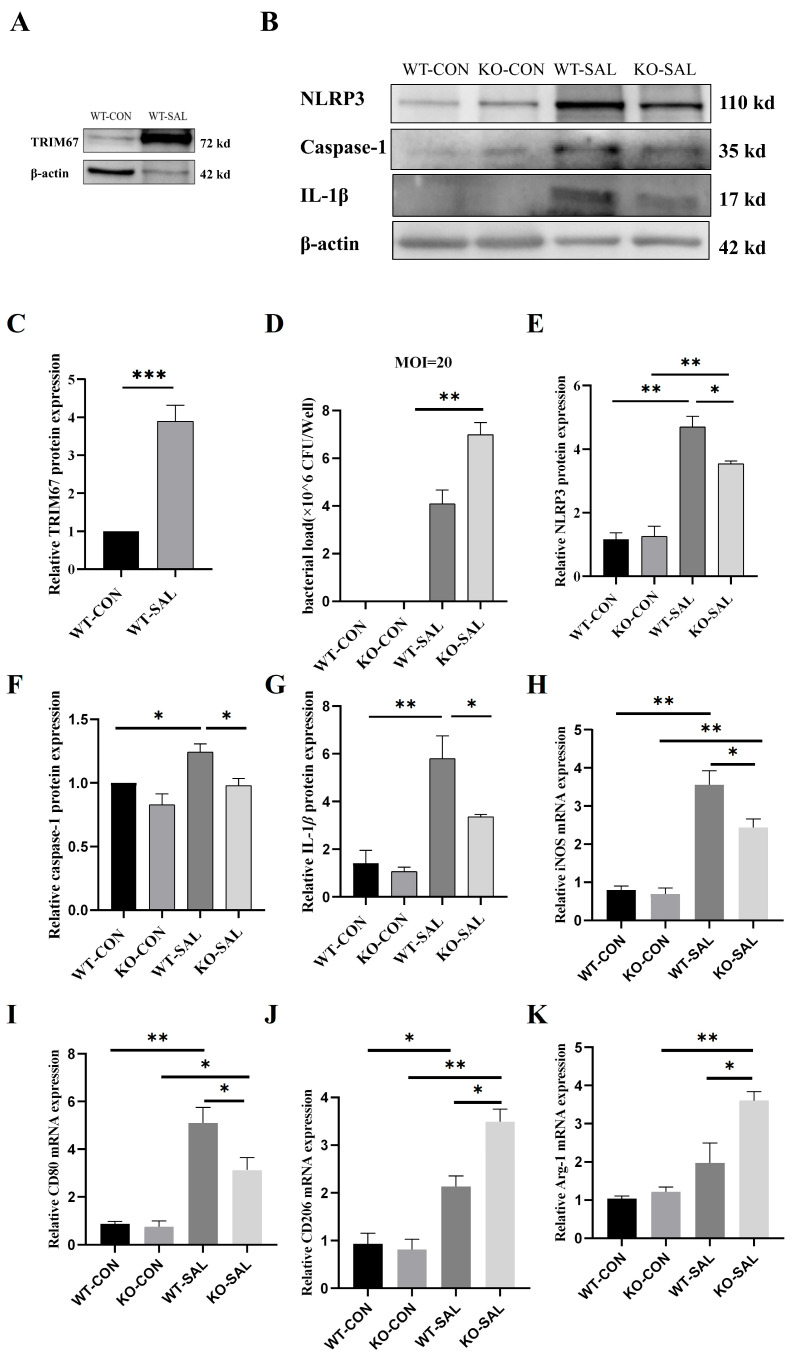
Impact of *TRIM67* knockout on macrophage polarization and NLRP3 inflammasome activation in PMs. (**A**) Western blot analysis of *TRIM67* protein expression in PMs; (**B**) Western blot analysis of NLRP3, caspase-1, IL-1β, and β-actin protein expression in PMs; (**C**) relative protein expression of *TRIM67* in PMs, *n* = 4; (**D**) bacterial load in PMs, *n* = 4; (**E**–**G**) relative protein expression of NLRP3, caspase-1 and IL-1β in PMs, *n* ≥ 3; (**H**,**I**) relative mRNA expression of M1 macrophage-related genes (iNOS and CD80) in PMs, *n* = 3; (**J**,**K**) relative mRNA expression of M2 macrophage markers (Arg-1 and CD206) in PMs, *n* = 3. * *p* < 0.05, ** *p* < 0.01 and *** *p* < 0.001.

Previous studies have demonstrated that *S.* Typhimurium infection significantly upregulates *TRIM67* expression in the intestine and MLNs of mice, and *TRIM67* knockout impairs bacterial clearance. Given the central role of macrophages in combating systemic *S.* Typhimurium infection, we isolated PMs from WT and KO mice to investigate whether *TRIM67* plays a role in macrophage-mediated clearance of *S.* Typhimurium. In vitro infection experiments revealed that *TRIM67* mRNA and protein levels were significantly elevated 1 h post-infection at an MOI of 20 (Figure 8A,C). By 12 h post-infection, bacterial loads in PMs from the KO group were significantly higher than in the WT group (Figure 8D), consistent with in vivo observations, indicating that *TRIM67* regulates macrophage-mediated *S.* Typhimurium clearance.

*TRIM67* knockout suppressed *S.* Typhimurium-induced NLRP3 inflammasome activation, as evidenced by significantly higher expression levels of NLRP3, caspase-1, and IL-1β in the WT-SAL group compared to the WT-CON, KO-CON, and KO-SAL groups (Figure 8B,E–G). Further studies revealed that *TRIM67* knockout inhibited M1 polarization of macrophages in MLNs during infection. qRT-PCR analysis showed that mRNA levels of M1 markers (iNOS and CD80) were significantly elevated in the WT-SAL and KO-SAL groups compared to the WT-CON and KO-CON groups (*p* < 0.01 or 0.05), but iNOS and CD80 expression in the KO-SAL group was significantly lower than in the WT-SAL group (*p* < 0.05). Similarly, mRNA levels of M2 markers (Arg1 and CD206) were significantly higher in the WT-SAL and KO-SAL groups compared to the WT-CON and KO-CON groups (*p* < 0.01 or 0.05), with Arg1 and CD206 expression in the KO-SAL group significantly exceeding that in the WT-SAL group (*p* < 0.05) (Figure 8H–K), confirming that *TRIM67* knockout inhibits M1 polarization.

## 4. Discussion

The TRIM protein family, as an important member of E3 ubiquitin ligases, plays a central role in host immune defense by regulating pattern recognition receptors, immune fitness molecules, and kinases associated with innate immune signaling pathways [39,40,41]. The present study reveals for the first time the key mechanism of *TRIM67* in host resistance to *S*. Typhimurium infection. After infection, the expression of *TRIM67* was significantly upregulated in mouse ileum, colon, mesenteric lymph nodes (MLNs), and peritoneal macrophages (PMs), suggesting its involvement in infection regulation. Bacterial loading experiments further confirmed that *TRIM67* deficiency impaired host clearance of Salmonella, as evidenced by accelerated weight loss and reduced survival in infected mice. This finding expands the functional spectrum of the TRIM family in infection control and contrasts with the mechanisms by which TRIM25 promotes Mycobacterium tuberculosis survival through the p38 MAPK/NF-κB pathway [42] and TRIM21 mediates increased macrophage death after Salmonella infection [43], highlighting the functional diversity of TRIM proteins.

The intestinal mucus barrier is the first line of defense against pathogen invasion, in which cuprocytes maintain the barrier function by secreting mucus [44]. In this study, we found that *S*. Typhimurium infection resulted in a significant reduction in the number of colonic cuprocytes, which was further exacerbated by *TRIM67* deficiency and accompanied by a severe disruption of the intestinal mucus barrier. This result echoes the role of *TRIM67* in maintaining intestinal homeostasis in obesity models [32], suggesting that its function is conserved across pathological scenarios. Notably, despite the reduced inflammatory cell infiltration in *TRIM67*-deficient mice, their structural intestinal damage was instead aggravated, presumably resulting in reduced pathogen clearance due to the weakened local immune response, which in turn triggered impaired digestive function. It was shown that *TRIM67* may maintain the intestinal barrier to inhibit *S*. Typhimurium colonization through a dual mechanism: direct promotion of cuprocyte survival and modulation of a modest early inflammatory response to repair damage.

The regulation of the NLRP3 inflammatory vesicle signaling pathway by the TRIM protein family has been widely reported [8,45,46]. In the present study, we found that intestinal NLRP3 expression was significantly upregulated after infection in wild-type mice, whereas *TRIM67* deletion inhibited this process, resulting in reduced caspase-1 activity and decreased pro-inflammatory cytokine release. This mechanism bears functional similarity to the pathway by which TRIM65 regulates NLRP3 activation through ubiquitination [47]. Inadequate activation of NLRP3 inflammatory vesicles will doubly impair host defense: on the one hand, it reduces pro-inflammatory factor release and delays neutrophil-mediated bactericidal responses; on the other hand, it allows for sustained proliferation of Salmonella in macrophages, which is consistent with an increase in bacterial MLN in *TRIM67* knockout mice load increase in the MLN of *TRIM67* knockout mice. This mechanism is highly consistent with previous reports on the role of NLRP3 inflammasome in limiting *S.* Typhimurium replication [48], suggesting that *TRIM67* may enhance early bactericidal activity of macrophages by positively regulating NLRP3 signaling, but the exact mechanism needs further investigation.

Macrophage phenotypic polarization is a key component of anti-infection immunity. In this study, we showed that *TRIM67* deficiency significantly inhibited the recruitment and expansion of macrophages after infection, although it did not affect the number of colonic macrophages at homeostasis. More importantly, *TRIM67* deficiency disrupts polarization homeostasis, inhibits M1-type (pro-inflammatory/bactericidal) phenotype switching, and impairs macrophage antimicrobial function. This finding contrasts interestingly with the regulatory mechanisms of other members of the TRIM family: e.g., TRIM59 deficiency promotes M1 polarization through the STAT1 pathway [49], TRIM24 deficiency enhances M2 polarization and impairs antitumor immunity [50], and Trim33 deficiency affects macrophage dynamics in colitis [51]. Studies suggest that *TRIM67* may regulate macrophage fate decisions through as yet unelucidated signaling nodes (e.g., the NLRP3 pathway or ubiquitination modification targets), and the specific mechanisms deserve to be explored in depth.

However, this study still has some limitations. Although most current macrophage typing statuses are mostly determined using the M1/M2 ratio, it has been suggested that M1/M2 is an oversimplification of macrophage subtypes [52,53]. In addition, this study used only a single animal model, the mouse, and lacks validation in clinical or primate settings. Future methods for macrophage typing need to be further optimized. The conclusions of this study need to be further explored in the clinic and in multiple animal models to explore TRIM67-targeted therapeutic approaches against Salmonella infections.

## 5. Conclusions

This study reveals the central role of *TRIM67* in the early stage of host resistance to *S.* Typhimurium intestinal infection: its deletion disrupts the intestinal mucus barrier, inhibits the activation of NLRP3 inflammatory vesicles, and disrupts the macrophage M1/M2 polarization balance, leading to a reduction in the early bactericidal capacity and persistent bacterial infection. This finding provides a new strategy for anti-gastrointestinal infection therapy: targeting *TRIM67* may promote the inflammatory response by modulating NLRP3 activity or break through the immune escape of drug-resistant bacteria by remodeling macrophage polarization, which opens up new avenues for the development of host-directed therapies.

## Figures and Tables

**Table 1:** Scoring criteria for inflammatory cell infiltration.

Score	Description
0	No inflammatory cell infiltration or minimal infiltration confined to the mucosal layer.
1	Increased inflammatory cells, continuously distributed in the lamina propria.
2	Significantly increased inflammatory cells, but not fully penetrating the submucosal layer.
3	Transmural inflammatory cell infiltration, involving the lamina propria, submucosa, and muscularis mucosa.

**Table 2:** Primers used in qRT-PCR.

Gene	Sequence (5′-3′)
β-actin	F: AGAGGGAAATCGTGCGTGAC
	R: CAATAGTGATGACCTGGCCGT
*TRIM67*	F: ACTCGGCAGAAAGCCAAGC
	R: CTGCTCTTGCGAGGTTTGC
NLRP3	F: TCCACAATTCTGACCCACAA
	R: ACCTCACAGAGGGTCACCAC
IL-1β	F: TCTTTGAAGTTGACGGACCC
	R: TGAGTGATACTGCCTGCCTG
Caspase-1	F: AAACACCCACTCGTACACGTCTTG
	R: AGGTCAACATCAGCTCCGACTCTC
Arg-1	F: CTCCAAGCCAAAGTCCTTAGAG
	R: GGAGCTGTCATTAGGGACATCA
iNOS	F: ACATCGACCCGTCCACAGTAT
	R: CAGAGGGGTAGGCTTGTCTC
CD206	F: CTCTGTTCAGCTATTGGACGC
	R: TGGCACTCCCAAACATAATTTGA
CD80	F: TCCAAGGCTCATTCT
	R: TTGTAACGGCAAGG

**Table 3:** Antibodies used in Western blot and Immunohistochemical Staining.

Antibody	Company	Catalog/Purpose and Dilution Ratio
β-Actin Rabbit mAb	ABclonal	AC026/WB 1:100,000
NLRP3 Rabbit mAb	ABclonal	A24294/WB 1:1000, IHC 1:200
Caspase-1/Cleaved Caspase-1	Wanleibio	WL03450/WB 1:1000
IL-1β Rabbit pAb	ABclonal	A1112/WB 1:1000
F4/80 Rabbit pAb	Bioss	bs11182R/IHC 1:500

## Data Availability

The original contributions presented in this study are included in the article/Supplementary Materials. Further inquiries can be directed to the corresponding authors.

## Supplementary Materials

The following supporting information can be downloaded at: https://www.mdpi.com/article/10.3390/microorganisms13061267/s1, Figure S1: Overview of targeting strategies to generate *TRIM67* knockout mice.; Figure S2: Genetic map; Figure S3: OD600 value—scatter plot of *S*. Typhimurium concentration; Figure S4: The strategy of constructing mouse models of *S*. Typhimurium infection; Figure S5: Validation of the *TRIM67* knockout mouse model. Figure S6: A complete gating strategy for flow cytometry.
