# Supplementary material for: The Impact of TRIM67 Knockout on Early Intestinal Antimicrobial Capacity in Mice Infected with Salmonella enterica serovar Typhimurium ATCC 14028
Source: Microorganisms. 2025 May 29;13(6):1267. doi: 10.3390/microorganisms13061267 (PMC12195278; doi:10.3390/microorganisms13061267)
Supplement: Supplementary file 1 [file microorganisms-13-01267-s001.zip › Supplements-microorganisms-3567567.pdf]

Supplement

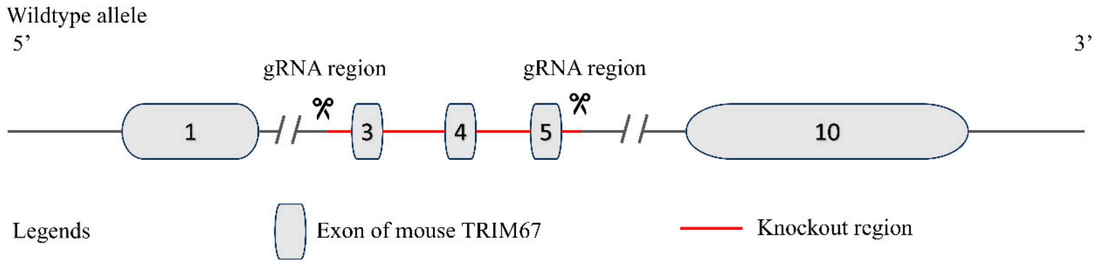

Supplementary Figure S1 Overview of targeting strategies to generate *TRIM67* knockout mice.

Knockdown of exons 3, 4, and 5 of *TRIM67* by CRISPR Cas9. Red lines indicate deleted regions.

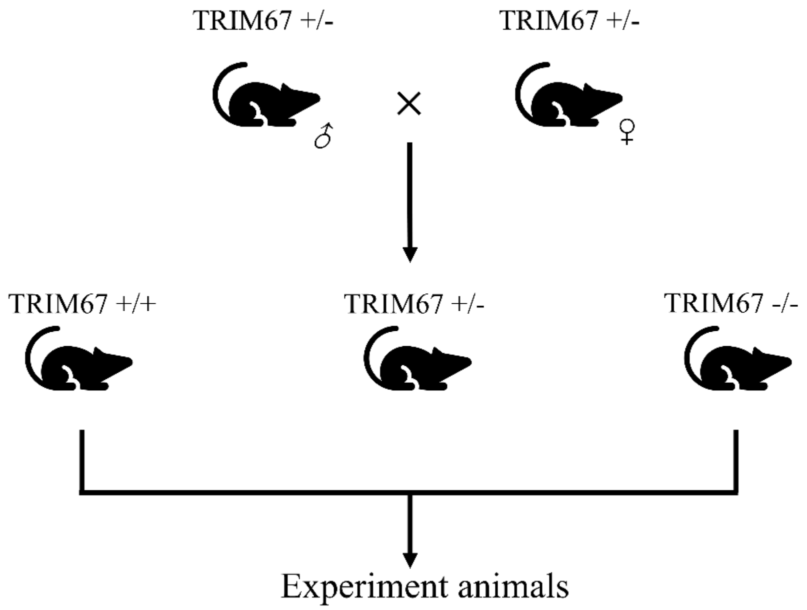

Supplementary Figure S2 Genetic map

TRIM67+/− mice were freely mated to produce offspring mice with different genotypes of *TRIM67*+/+, *TRIM67*+/−, and *TRIM67*−/−. The purebred *TRIM67*+/+ and *TRIM67*−/− in the offspring were used as test animals in this study.

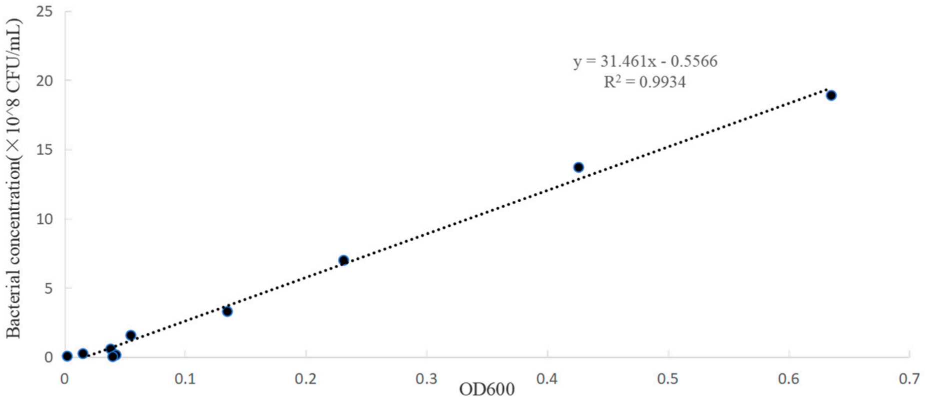

Supplementary Figure S3 OD600 value - scatter plot of *S*. Typhimurium concentration

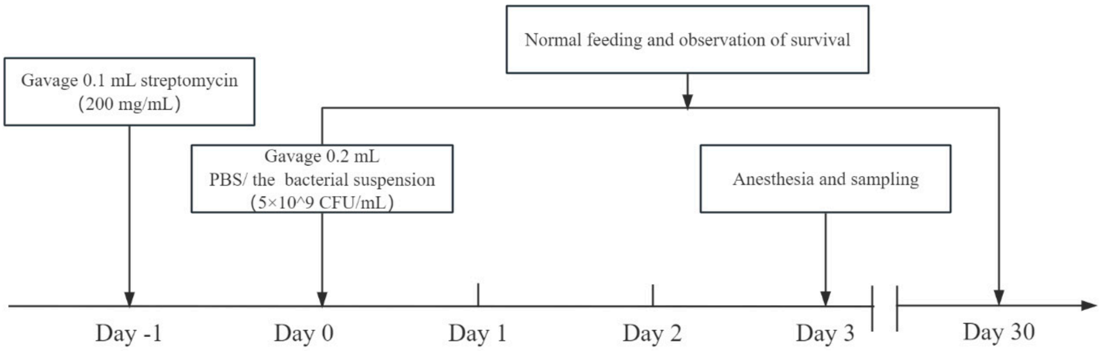

Supplementary Figure S4 The strategy of constructing mouse models of *S*. Typhimurium infection

The day before infection (Day -1), all mice were fasted from food and water for 4 hours. Both control (CON) and infected (SAL) groups were orally administered 0.1 mL (200 mg/mL) of streptomycin by gavage. Food and water intake was resumed 4 hours after administration. At the same time on the next day (Day 0), the mice were again fasted for 4 hours. The control group (CON) was then gavaged with 0.2 mL of sterile PBS, while the infected group (SAL) was gavaged with 0.2 mL of the bacterial suspension (5×10^9 CFU mL^{-1}). Some mice were used to observe survival for 30 days post-infection (Day 30), and some mice were anesthetized and sampled 3 days after infection (Day 3).

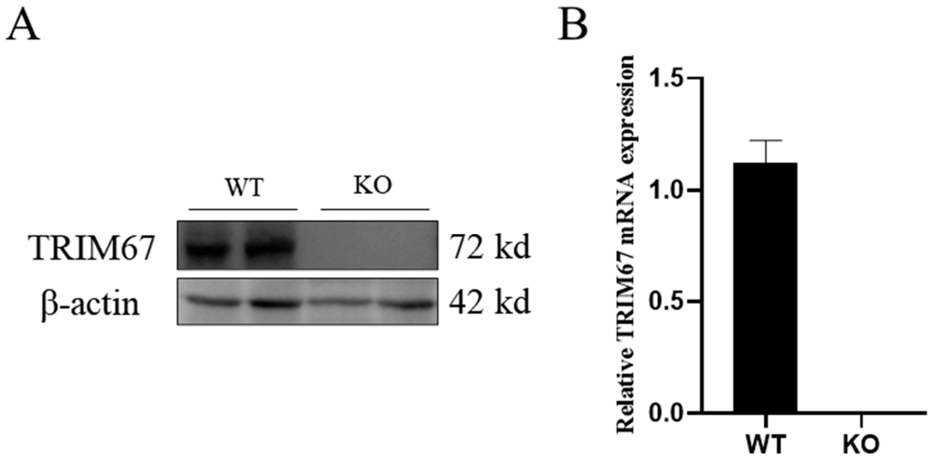

Supplementary Figure S5 Validation of the *TRIM67* knockout mouse model

(A) Protein immunoblotting of *TRIM67* within the mouse MLN. (B) Relative mRNA expression of *TRIM67* within the mouse MLN, *n*=4

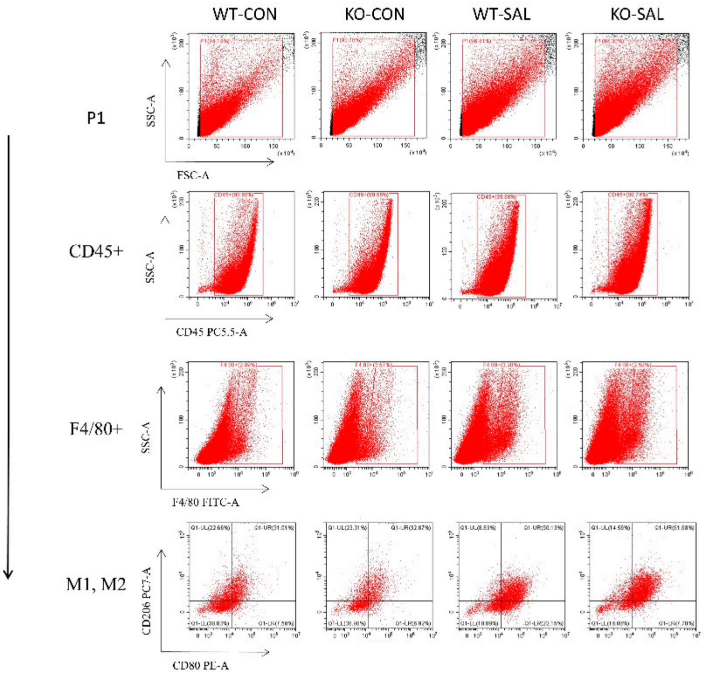

Supplementary Figure S6 A complete gating strategy for flow cytometry
